# Supplementary material for: Physics-informed neural networks for solving nonlinear diffusivity and Biot’s equations
Source: PLoS One. 2020 May 6;15(5):e0232683. doi: 10.1371/journal.pone.0232683 (PMC7202655; doi:10.1371/journal.pone.0232683)
Supplement: Attachment — Submitted filename: review.pdf [file pone.0232683.s001.pdf]

Review of: Physics-informed Neural Networks for Solving Nonlinear Diffusivity and Biot's equations, PONE-D-19-24031

Summary of Content

The authors aim to indirectly estimate the matrix properties of porous media through an approximation of the associated inverse problem by neural networks. They propose point-to-point multilayer perceptrons for solving both inverse and forward problems of Biot's equation, equipped with an advanced cost function that incorporates the local residual. They conduct a range of sensitivity studies and show that for the chosen academic problems, good NN approximations can be found.

General remarks:
The general idea of the paper is interesting and worth investigating. However, the quality and depth of the presentation is lacking. I have listed all my detailed concerns below. The most pressing concerns I have are:

- The paper lacks insights and conclusions. It reads a lot like a work package description: First we did then, then this, then this. Why things are done, how results are critically judged and how they can be interpreted is missing in many cases.
- Many important details are either not mentioned or not well explained. The most striking example is the PINN approach, which the authors claim to follow: While some effort is spent on explaining what the modified cost functions for the PINN network are, the most important aspect of PINNs, that is the evaluation of the residual function within the context of the NN itself, is not mentioned at all. This leaves the reader with the impression that the PINN approach is not followed, instead, the authors merely have augmented the cost function. This lack of detail here and at other places (see detailed comments below) makes the work hard to evaluate.
- There appears to be no splitting of the data into training, validation and test set, as is common to avoid loss of generalization and tainting of test set.
- The authors make the assumption that the same hyperparameters are valid for the forward and inverse problem without justification. This needs to be investigated, discussed and justified.
- The chosen demonstration cases are very simple analytical functions, even constants of one for the inverse problem. I would like to see an application to a more complex situation. The authors claim that their goal is to beat traditional simulation approaches in terms of speed. Why not show this for a relevant, non-trivial case?

The style, grammar and clarity of the paper are acceptable, but could definitely be improved.

Detailed and specific remarks:

- Abstract: "crucial to indirect estimate the": should be: indirectly
- l9: what about other discretization methods, e.g. finite differences, discontinuous Galerkin schemes...
- l11: While this statement might be true, the authors do not discuss the costs associated with the PINN approach in this work, so the issue whether NN based inverse problem solutions can be efficient is not answered in this paper.
- l16: this is only one side of the issue: In many areas of application, the data is already there or readily available and just needs to be harvested.
- l19: please also include citations from NNs used in fluid dynamics that do not rely on PINN. There are quite a few, for example in the field of RANS or LES closure with NNs.
- l39: check double commas
- l55: should be "the total stress"
- l59: replace comma by "and"
- l73: please explain the reasoning and consequences behind this simplification.
- l75: lowercase: results
- Please define either here or later the forward and inverse problems, including knowns and unknowns, precisely for readers not intimately familiar with your area of application.
- l77-l88: please include a discussion on point-wise vs. stencil-wise learning / approximation. In non-linear problems like Biot's law, interactions are typically non-local, thus, an input stencil would make sense. Please explain your reasoning for using point-to-point learning only.
- Fig 1.: This is a standard MLP net. Please also cite some of the groundbreaking early work by Hinton on this.
- The basic concepts of the PINN approach are not well explained. They should at least be summarized in a short paragraph. Also,it remains unclear if the authors actually compute the constraints by differentiating the NN itself (as is done in the original PINN papers), or if the evaluation of the constraint is done otherwise. The authors should clearly state how this done in their work. Is the network differentiated and thus optimized to enforce the constraints, or is it just enforced through the cost function?
- l93, Eq. 15: This is not a constraint, but a function. The constraint should include the =0 part.
- l111: explain the reasoning for the different sample positions for the forward and inverse problems.

- l112: Where does the training data come from? Analytical solutions? Numerical simulation? Please also provide at least a basic statistical analysis of the training data.
- Please provide information on the training of the NNs themselves, including hardware used, software used, regularization, step sizes, training time per epoch and other statistics that are useful.
- l107: The MSE on the training data and the PINN constraint are probably very different, at least there is no reason to assume that they are on the same level / order of magnitude. How is this accounted for in the loss function? It seems that both errors are just linearly added.
- l137: What is the overall domain size?
- l140: What is meant by "uniformly" in this context?
- l140: Why is scikit-learn mentioned here specifically? The split into train / val / test sets does not require a special package. It is strange that this specific detail is given here, while the rest of the technical details on the net are left out
- l140: So is there no validation set, i.e. the hyperparameter tuning and the final evaluation are done on the test set? This is not a common practice, as it allows information bleeding from the test set into the training, risking generalization loss. The authors should repeat their investigations by splitting the data into the three buckets, and then perform NN design and training on the train/val datasets and finally report the results from blind application to the test set!
- l141: Why is the test set enormously large compared to the training set?
- l146: How is the sensitivity analysis conducted? Brute force? How are the sampling points in the hyperparameter space for the analysis chosen?
- l152: This statement is very vague at best. Clearly, the forward and inverse mapping to be learned by the NN are not similar - why should they be? Even if parts of the cost function look similar, the mapping is completely different. They might be an inverse of one another in a sense, but this is still an open question. Speculating that hyperparameters are transferable is wrong even for different networks for the same (forward) problem, so I do not see any sound justification for doing it for the inverse. The authors should back up their claim with data / citations / proof. For the toy problems considered here where the inverse field can be prescribed exactly, the authors should investigate this before making this far-reaching assumption.
- l155: Why this optimization algorithm? What are its advantages? Why full batch approach?
- l157: The listing of these steering parameters of the implementation without even explaining what they are does not make any sense. Either explain them properly and how they relate to the algorithm, or remove / move to

appendix. Also, how are local minima avoided / detected during training?

- l166: Have you tried adaptive sampling based e.g. on local variance?
- Please report the MSE for the data and PINN part separately, i.e. the two contributions as well as the overall MSE. Please also show the cost history for both errors.
- Tbl1: For most cases, the error drastically increases from 80 to 160 collocation points. What goes on here?
- l178: to lose $\neq$ to loose
- Tbl2: How long were the networks trained for? Same no of epochs or same no of steps?
- l176: How does this tbl. show overfitting? Take the last row for example: Increasing the neurons per layer does not lead to consistently worse results, in fact, the results improve up to $N_n = 40$. For $N_{hl} = 6$, the error drops again for higher N_n. This table does not show what the authors claim it does. It requires at least a thorough discussion.
- Eq. 33,34,35: Consider removing
- l194: So the unknowns are just constant in the whole field?
- l198: Compared against what?
- Fig4: "accuracy" should be accurate.
- Comment on why the prediction improves drastically for noisy data in some cases ($\phi_1, N_{tr} = 100$). Is the noise here actually preventing overfitting? please provide the cost history of training and validation data for theses cases.
- l211: So what are your conclusions from the previous section?
- l215: should be 2d not 2D
- l230-l236: This reads like a copy from the previous section, consider rewriting and see comments above.
- l243: How and why are the optimizers combined?
- How is overfitting estimated and avoided in all the cases shown?
- Tbl4, last column: Is overfitting happening here? What type of countermeasures were chosen?
- l280: Please show the PINN network, and please clarify if this is indeed a PINN approach, or an advanced cost function approach.
- l303ff: Why not scale them accordingly?
- tbl 6: The difficulty of training the parameters θ seems to vary greatly. Why are some much easier to approximate than others?
- l317: What is meant by CPU processing time? Computing the exact solutions? Training the network? deployment? How fast is the deployed network compared to the FV / FE solver the authors aim to beat?

- l316: ”an” instead on ”en”
- l318: What means ”usually scalable”?

In summary, the application of NNs to the inverse problem presented here is interesting. The paper however lacks sufficient detail and diligence to judge its usefulness and soundness. The authors should rework their manuscript, addressing the issues raised here and restructure their work accordingly to present a clear message. I recommend a major revision.
