# Supplementary material for: Physics-informed neural networks for solving nonlinear diffusivity and Biot’s equations
Source: PLoS One. 2020 May 6;15(5):e0232683. doi: 10.1371/journal.pone.0232683 (PMC7202655; doi:10.1371/journal.pone.0232683)
Supplement: Attachment — Submitted filename: plosOne_reply_to_reviewers0802.docx [file pone.0232683.s002.docx]

The authors thank and appreciate the editor and reviewer for their constructive comments.

Below we have for each comment inserted our response and described the changes made in the revised manuscript.

Main comments

The paper lacks insights and conclusions. It reads a lot like a work package description: First we did then, then this, then this. Why things are done, how results are critically judged and how they can be interpreted is missing in many cases

Reply: We have revised our manuscript according to the comments to give this additional information, see below.

Many important details are either not mentioned or not well explained. The most striking example is the PINN approach, which the authors claim to follow: While some effort is spent on explaining what the modified cost functions for the PINN network are, the most important aspect of PINNs, that is the evaluation of the residual function within the context of the NN itself, is not mentioned at all. This leaves the reader with the impression that the PINN approach is not followed, instead, the authors merely have augmented the cost function. This lack of detail here and at other places (see detailed comments below) makes the work hard to evaluate.
Reply: We have added ‘Training the PINN’ section, please find it from lines 144 to 193.

There appears to be no splitting of the data into training, validation and test set, as is common to avoid loss of generalization and tainting of test set

Reply: We now split our data into training, validation, and test sets accordingly and show the performance on the validation set and test sets are comparable.

The authors make the assumption that the same hyperparameters are valid for the forward and inverse problem without justification. This needs to be investigated, discussed and justified.

Reply: We have addressed this issue. Please find more detail in the ‘Detailed and specific remarks’ below.

The chosen demonstration cases are very simple analytical functions, even constants of one for the inverse problem. I would like to see an application to a more complex situation. The authors claim that their goal is to beat traditional simulation approaches in terms of speed. Why not show this for a relevant, non-trivial case?

Reply: The aim of this paper is to demonstrate the possibility of applying PINN to multiphysics problems, especially in the case of Biot’s equations. Therefore, we focus on simple examples, in which we can investigate the PINN behavior. We will then progress to more complex cases in our future studies. Besides, we do not aim in this paper to explore or conclude which methods are the best. The main aim is to investigate whether the PINN based method could serve as an alternative.

Detailed and specific remarks:

Abstract: "crucial to indirect estimate the": should be: indirectly

Reply: This has been changed.

l9: what about other discretization methods, e.g. finite differences, discontinuous Galerkin schemes...

Reply: We have revised and added more references pointing to finite difference and different types of finite element methods (mixed formulation, DG, EG) – lines 8 - 12.

l11: While this statement might be true, the authors do not discuss the costs associated with the PINN approach in this work, so the issue whether NN based inverse problem solutions can be efficient is not answered in this paper.

Reply: We have revised and added more references, please find them in lines 12- 16. Also see the comments just above.

l16: this is only one side of the issue: In many areas of application, the data is already there or readily available and just needs to be harvested.

Reply: We have reformulated accordingly – lines 22-24.

l19: please also include citations from NNs used in fluid dynamics that do not rely on PINN. There are quite a few, for example in the field of RANS or LES closure with NNs.

Reply: We have revised and added references, please find them in lines 25 - 28.

l39: check double commas

Reply: Done.

l55: should be "the total stress"

Reply: Done.

l59: replace comma by "and"

Reply: Done.

l73: please explain the reasoning and consequences behind this simplification.

Reply: We have revised and added two references, please find them in lines 78 - 80.

l75: lowercase: results

Reply: Done.

Please define either here or later the forward and inverse problems, including knowns and unknowns, precisely for readers not intimately familiar with your area of application.

Reply: We have added lines 218 – 220 (diffusivity) and lines 353 – 357 (Biot).

l77-l88: please include a discussion on point-wise vs. stencil-wise learning / approximation. In non-linear problems like Biot's law, interactions are typically non-local, thus, an input stencil would make sense. Please explain your reasoning for using point-to-point learning only.

Reply: We have added the discussion from lines 105 to 107.

Fig 1.: This is a standard MLP net. Please also cite some of the groundbreaking early work by Hinton on this.

Reply: We have added the references - line 85 and fig. 1.

The basic concepts of the PINN approach are not well explained. They should at least be summarized in a short paragraph. Also,it remains unclear if the authors actually compute the constraints by differentiating the NN itself (as is done in the original PINN papers), or if the evaluation of the constraint is done otherwise. The authors should clearly state how this done in their work. Is the network differentiated and thus optimized to enforce the constraints, or is it just enforced through the cost function?

Reply: We have now added a more thorough description of the principles of PINN from lines 97 to 107, 129 to 143, 145 to 193, 229 to 239 (diffusivity), and lines 359 to 363 (Biot).

l93, Eq. 15: This is not a constraint, but a function. The constraint should include the =0 part.

Reply: We have now clearly pointed out that these functions according to the differential equations ideally should be zero, which then represent the constraints. Accordingly, they are added as regularization terms in the cost function. We have added a clarification from lines 119 to 120 and 145 to 193.

l111: explain the reasoning for the different sample positions for the forward and inverse problems.

Reply: we have added our explanations from lines 132 to 143, 229 to 239 for the diffusivity equation and from lines 353 to 357 for the Biot’s equations.

l112: Where does the training data come from? Analytical solutions? Numerical simulation? Please also provide at least a basic statistical analysis of the training data.

Reply: We draw our training, validation, and test examples from the analytical solutions (Eq. 24 for the diffusivity equation and Eqs. 36 and 37 for the Biot’s case). In a real application, the examples would for the inverse cases be obtained from measurements. We added an explanation from lines 211 to 217 (diffusivity) and lines 346 to 352 (Biot).

Please provide information on the training of the NNs themselves, including hardware used, software used, regularization, step sizes, training time per epoch and other statistics that are useful.

Reply: all simulations are run on an XeonE5\_2660v3 processor with a single thread, as mentioned from lines 459 to 464. We build and train the neural networks using the Tensorflow platform, as mentioned from lines 107 to 108. The stopping criterion used is mentioned from lines 222 to 225. Training time depends on a number of examples and network architecture; we provide some examples from lines 459 to 464.

l107: The MSE on the training data and the PINN constraint are probably very different, at least there is no reason to assume that they are on the same level / order of magnitude. How is this accounted for in the loss function? It seems that both errors are just linearly added.

Reply: One could indeed have had a λ weight coefficient to regulate the impact of the two terms. We kept the coefficient of ½ as in the original PINN paper. As long as both term converges steadily towards zero, the exact value of λ is not of big importance. We have now inserted figures to illustrate how the two terms decrease individually. We both explore the case for the nonlinear diffusivity equation (Fig. 5) and the case of the nonlinear Biot’s equations (Fig. 9).

l137: What is the overall domain size?

Reply: It has been stated in the manuscript; line 203 for the diffusivity equation and line 327 for the Biot’s equations.

l140: What is meant by "uniformly" in this context?

Reply: We have clarified from lines 211 to 217 and 346 to 352.

l140: Why is scikit-learn mentioned here specifically? The split into train/ val / test sets does not require a special package. It is strange that this specific detail is given here, while the rest of the technical details on the net are left out

Reply: This is true. We have now left out this detail and instead, given more information on the other more relevant technical parameters.

l140: So is there no validation set, i.e. the hyperparameter tuning and the final evaluation are done on the test set? This is not a common practice, as it allows information bleeding from the test set into the training,risking generalization loss. The authors should repeat their investigations by splitting the data into the three buckets, and then perform NN design and training on the train/val datasets and finally report the results from blind application to the test set!

Reply: This is, of course, the correct procedure. We have now included validation and test sets. Please refer to lines 211 to 217 and 346 to 352.

l141: Why is the test set enormously large compared to the training set?

Reply: We have generated a lot of ground truth examples from an analytic solution. Because we only need “few” training examples to obtain a decent NN solution, we have a lot of remaining examples that are available for testing/validation.

146: How is the sensitivity analysis conducted? Brute force? How are the sampling points in the hyperparameter space for the analysis chosen?

Reply: The sensitivity analysis preferably should cover the region in the hyperparameter space from which we can identify a minimum of the loss function. The parameters corresponding to this minimum is then likely to ensure adequate generalization capabilities of the network. We also added a clarification to our manuscript from lines 265 to 268.

l152: This statement is very vague at best. Clearly, the forward and inverse mapping to be learned by the NN are not similar - why should they be? Even if parts of the cost function look similar, the mapping is completely different. They might be an inverse of one another in a sense, but this is still an open question. Speculating that hyperparameters are transferable is wrong even for different networks for the same (forward) problem, so I do not see any sound justification for doing it for the inverse. The authors should back up their claim with data / citations / proof. For the toy problems considered here where the inverse field can be prescribed exactly, the authors should investigate this before making this far-reaching assumption.

Reply: As now explained from lines 183-193 in the revised text, the mappings to be learned by the NNs are very similar, because in both cases the mapping that the NN should learn, is to map the (x,t) input space to the (u,p) output space and at the same time the loss functions are identical – (the PI terms with regard to boundary and initial conditions are however not used for the inverse case). The only conceptual and essential difference is that the physical parameters \theta are unknown in the case where we address the inverse problem. Since the unknown \theta parameters, however, are adjusted along with the conventional weights and biases in the NN during training, the idea is that these parameters converge to their true values by training the network to predict u and p. But still, the training is about learning the mapping from (x,t) to (u,p) so the mappings are very much alike for the forward and the inverse case.

Put in another way, we do not have NN for the inverse case that actually is trained to minimize predicted \theta-values relative to ground truth ones. This would also require that we had a training set where each training example coupled one value of \theta (which then would be the output value) to a number of coupled values of (x,t,u,p) (which then would represent a given representation of the input space).

The above explanation is the reason why the hyperparameters estimated to work for the forward problem is a qualified estimate to use also for the inverse problem. Since the optimum hyperparameters, in general, will be dependent on the number of training examples a reasonable requirement will be that the number of training examples used when training the network for the inverse problem is similar to the number of examples used in the forward problem when estimating the hyperparameters. As by definition, we do not know the true value of \theta for the inverse case, so we further have to assume that the exact values of \theta have a limited influence on the hyperparameters. A more direct way of estimating the hyperparameters in the inverse case would be to divide the amount of measured data into training and validation and then select the hyperparameters that minimize the MSE term with regard to u and p. The downside of this would be that we could not spend all the measurement data on the specific training cycle used to estimate theta.

We have now included a Table 3, which together with lines 293 to 300 verify that the hyperparameters selected from the forward problem match the hyperparameters found by applying the “direct” approach just described. We have done similarly for the Biot’s equations, see Table 7 and lines 434 to 440.

l155: Why this optimization algorithm? What are its advantages? Why full batch approach?

Reply: We elaborate this from lines 225to 227.

l157: The listing of these steering parameters of the implementation without even explaining what they are does not make any sense. Either explain them properly and how they relate to the algorithm, or remove / move to appendix. Also, how are local minima avoided / detected during training?

Reply: We agree the listing of these parameters is not meaningful. We have now deleted them. Concerning local minima, we cannot guarantee to avoid them. However, we have now run the training cycles using different initial seeds to study the variation and average our results over many realizations. Our tables now contain these averages aiding in illustrating the behavior of the network for different choices of hyperparameters and the amount of training examples. Please find the revised text from lines 251 to 258.

l166: Have you tried adaptive sampling based e.g. on local variance? Please report the MSE for the data and PINN part separately, i.e. the two contributions as well as the overall MSE. Please also show the cost history for both errors.

Reply: For the current work, we only used the Latin-hypercube method. We will try adaptive sampling-based methods in our future work. Moreover, we have now reported the MSE for the data and PINN separately for one case of the nonlinear diffusivity equation (Fig. 5) and one case of the nonlinear Biot’s equations (Fig. 9).

Tbl1: For most cases, the error drastically increases from 80 to 160 collocation points. What goes on here?

Reply: In our original draft, the results in the Table were based on only one training cycle for each combination in the Table (i.e., with a given random seed). For that reason, it was difficult to see the trend. We have now included tables where we average over 3 and 24 realizations, respectively, to better illustrate the trend of lower loss with more training examples. We also have added the clarification of this behavior from lines 235 to 246. We have done the same thing with Biot’s equations - Tab 5.

l178: to lose 6= to loose

Reply: Done

Tbl2: How long were the networks trained for? Same no of epochs or same no of steps?

Reply: We have added a clarification from lines 202 to 205.

l176: How does this tbl. show over fiitting? Take the last row for example: Increasing the neurons per layer does not lead to consistently worse results,in fact, the results improve up to Nn = 40. For Nhl = 6; the error drops again for higher Nn. This table does not show what the authors claim it does. It requires at least a thorough discussion.

Reply: We agree that the Table in the draft was not convincing inillustrating overfitting. As we have now included Tables 1, 2, 5, 6 (Panels B-C) that present averages of the error term over a number of training realizations, the overfitting should now be evident from the presented Tables. Please find Table 2 and lines 264 – 275. We also have included the clarification of the Biot’s equations section Table 6 and lines 406 to 413.

Eq. 33,34,35: Consider removing

Reply: We have removed Eq. 33; however, Eqs. 34 (now it is 33) and 35 (now it is 34) are essential to define our loss function.

l194: So the unknowns are just constant in the whole field?

Reply: Yes. We also added more clarification for both diffusivity and Biot’s equations. For this paper, we assume all physical parameters are constant throughout the domain. The heterogeneous cases will be our next target.

l198: Compared against what?

Reply: Since we do not compare the precision with that of another technique, we have changed the formulation. We have revised our text accordingly in lines 306 to 312, for example.

Fig4: "accuracy" should be accurate.

Reply: done

Comment on why the prediction improves drastically for noisy data in some cases (_1;Ntr = 100). Is the noise here actually preventing overfitting? please provide the cost history of training and validation data for these cases.

Reply: The table in the original submission did not show average performances over several realizations, and this is the reason one could observe “drastically” changes. So the trend of decreased accuracy with increasing noise was partially buried in stochastic variations. We have now revised this table to be an average over several realizations, see Table 4 (diffusivity) and Table 8 (Biot). One can now easily spot the expected trend of the percentage error. In addition, we have also added error bars in Fig 6 (for the noise-free case) to illustrate the stochastic variation. Fig 6. shows that when the number of training examples is small the performance varies significantly (large standard deviation). As the number of training examples increases the standard deviation decreases accordingly. Please find the clarification for the diffusivity equation from lines 319 to 321. We also have done the same thing for Biot’s equations; see Tab. 8 and Fig. 10 and lines 454 to 458.

l211: So what are your conclusions from the previous section?

Reply: We have added lines 325 to 328.

l215: should be 2d not 2D

Reply: Done

l230-l236: This reads like a copy from the previous section, consider rewriting and see comments above.

Reply: We have now rewritten lines 346 to 357.

l243: How and why are the optimizers combined?

Reply: We have now added explanations from lines 365 to 377.

How is overfitting estimated and avoided in all the cases shown?

Reply: We have now addressed this topic from lines 144 to 193 and also shown the experiment in Tables 2, 3, 6, and 7.

Tbl4, last column: Is overfitting happening here? What type of countermeasures were chosen?

Reply: The former Table 4 is now occurring as Table 5 in the revised manuscript and now includes average values over several training cycles. As also addressed above, this now helps in illustrating that the performance increases with more training examples.

l280: Please show the PINN network, and please clarify if this is indeed a PINN approach, or an advanced cost function approach.

Reply: This subject has now been addressed from lines 97 to 108 and lines 145 to 193. (see also above comments).

l303_: Why not scale them accordingly?

Reply: Sorry, we did not understand this comment.

tbl 6: The difficulty of training the parameters _ seems to vary greatly. Why are some much easier to approximate than others?

Reply: As we illustrated in Fig. 10, as we average over many realizations, the error-levels for the different physical parameters become comparable. Former Table 6 is now replaced with Table 8 and now shows average values over several runs.

l317: What is meant by CPU processing time? Computing the exact solutions? Training the network? deployment? How fast is the deployed network compared to the FV / FE solver the authors aim to beat?

Reply: We agree with the reviewer, we have now explicitly stated that the CPU-time is with regard to the training time of the neural network. See lines 459-464. In the paper, the aim is not to compare the PINN to other techniques with respect to computational time, but to explore whether PINN could be an alternative way to the traditional solvers. Anyway, we chose to give an example of the CPU-time as a way to convey the practicality of applying the methodology.

l316: "an" instead on "en"

Reply: done

l318: What means "usually scalable"?

Reply: We agree that this is an imprecise formulation. We have now removed the sentence from the manuscript.

In summary, the application of NNs to the inverse problem presented here is interesting. The paper however lacks sufficient detail and diligence to judge its usefulness and soundness. The authors should rework their manuscript, addressing the issues raised here and restructure their work accordingly to present a clear message. I recommend a major revision.
